# Serum ferritin levels in non-anemic children and adolescents with attention-deficit/hyperactivity disorder: associations with clinical presentations and symptom severity

**DOI:** 10.3389/fpsyt.2026.1875919

**Published:** 2026-08-07

**Authors:** Burak Kamış, Şule Çalışkan Kamış, Cumali Yüksekkaya, Sema Aktepe Vural, Serkan Güneş

**Affiliations:** 1 Department of Child and Adolescent Psychiatry, University of Health Sciences, Adana Faculty of Medicine, Adana City Training and Research Hospital, Adana, Türkiye; 2 Department of Pediatric Hematology and Oncology, University of Health Sciences, Adana Faculty of Medicine, Adana City Training and Research Hospital, Adana, Türkiye

**Keywords:** ADHD, adolescents, children, Conners Rating Scale, ferritin, hyperactivity, iron metabolism

## Abstract

**Background:**

Attention-deficit/hyperactivity disorder (ADHD) is a common neurodevelopmental disorder in childhood and adolescence. Iron is involved in dopamine synthesis, myelination, and brain energy metabolism; therefore, reduced iron stores may be relevant to ADHD, although serum ferritin findings remain inconsistent.

**Objective:**

This study aimed to compare serum ferritin levels between non-anemic children and adolescents with ADHD and controls, and to evaluate their association with ADHD clinical presentations and symptom severity.

**Methods:**

This retrospective cross-sectional analytical study included 105 children and adolescents with DSM-5-TR ADHD and 102 age-comparable controls without known psychiatric or chronic systemic disease, recruited from pediatric outpatient clinics during the same period. Participants with anemia, active systemic disease, intellectual disability, or laboratory evidence of acute inflammation were excluded. Hemoglobin, ferritin, and C-reactive protein (CRP) levels were compared between groups. Same-day ferritin/CRP values from the same institutional laboratory were used. Serum iron samples were obtained in the morning after fasting. Within the ADHD group, iron-related parameters, ADHD presentation, pharmacotherapy/supplementation status, and parent- and teacher-rated Conners scores were evaluated. Non-parametric tests, Spearman correlation analysis, and multivariable logistic regression were performed.

**Results:**

The groups were comparable in age, hemoglobin, and CRP. Male sex was more frequent in the ADHD group [77/105 (73.3%) vs. 49/102 (48.0%); p<0.001]. Serum ferritin levels were lower in the ADHD group [15.10 ng/mL, interquartile range (IQR): 11.10–20.60 vs. 20.10 ng/mL, IQR: 15.03–28.33; p<0.001]. After adjustment for age, sex, hemoglobin, and CRP, higher ferritin levels were associated with lower odds of ADHD (OR = 0.927, 95% CI: 0.895–0.961; p<0.001). Ferritin levels differed across ADHD presentations and showed weak negative correlations with parent- and teacher-rated hyperactivity scores.

**Conclusion:**

Non-anemic children and adolescents with ADHD had lower serum ferritin levels than controls. Ferritin may be a supportive biological parameter in selected ADHD subgroups, but not a diagnostic or severity biomarker.

## Introduction

Attention-deficit/hyperactivity disorder (ADHD) is one of the most common neurodevelopmental disorders in childhood and adolescence. It is characterized by developmentally inappropriate and impairing levels of inattention, hyperactivity, and impulsivity, as defined in the Diagnostic and Statistical Manual of Mental Disorders, Fifth Edition, Text Revision (DSM-5-TR) (1). Recent evidence indicates that ADHD has a worldwide prevalence of approximately 8% among children and adolescents, although prevalence estimates may vary according to methodology, diagnostic criteria, informant source, and population characteristics (2, 3).

The etiology of ADHD is multifactorial and involves complex interactions between genetic susceptibility, neurodevelopmental mechanisms, environmental exposures, and neurobiological alterations. Dysregulation of dopaminergic and noradrenergic neurotransmission has long been considered central to the pathophysiology of ADHD, particularly in relation to executive functioning, reward processing, motor activity, and attentional control (4, 5).

Iron is an essential micronutrient involved in several neurobiological processes, including dopamine synthesis, myelination, mitochondrial energy metabolism, and neuronal development. It acts as a cofactor for tyrosine hydroxylase, the rate-limiting enzyme in dopamine synthesis. Therefore, reduced iron stores may contribute to alterations in dopaminergic transmission, providing a biologically plausible link between iron status and ADHD-related symptoms (6, 7). Importantly, these neurobiological effects may occur even in the absence of overt anemia, suggesting that iron stores may be clinically relevant beyond hemoglobin levels alone.

Serum ferritin is widely used as a peripheral marker of iron stores. However, ferritin is also an acute-phase reactant and may be influenced by inflammation, infection, chronic disease, nutritional status, and supplementation. These factors may partly explain the inconsistent findings reported in studies examining ferritin levels in children and adolescents with ADHD (7). Therefore, studies evaluating ferritin in ADHD should consider hemoglobin status and inflammatory markers such as C-reactive protein (CRP) when interpreting serum ferritin levels.

Previous systematic reviews and meta-analyses have suggested that children with ADHD may have lower serum ferritin levels than controls, whereas findings for serum iron and other iron-related parameters are less consistent (8, 9). However, individual studies have reported conflicting results. While Okuyucu et al. reported lower ferritin levels and associations between ferritin and symptom-related outcomes in children with ADHD, Percinel et al. found no significant difference in ferritin levels between children and adolescents with ADHD and controls (10, 11).

ADHD is clinically heterogeneous. According to DSM-5-TR, ADHD may present as predominantly inattentive, predominantly hyperactive/impulsive, or combined presentation (1). In clinical practice, symptom severity and functional impairment are commonly assessed using parent- and teacher-rated scales in addition to clinical evaluation (12, 13). Therefore, examining whether ferritin levels are associated not only with ADHD diagnosis but also with clinical presentation and symptom severity may help clarify the potential role of iron metabolism in ADHD. Nevertheless, ferritin should be interpreted as a potential supportive biological parameter rather than as a diagnostic marker, given the multifactorial nature of ADHD and the peripheral nature of serum ferritin measurement.

The primary aim of this study was to compare serum ferritin levels between non-anemic children and adolescents with ADHD and a control group without known psychiatric or chronic systemic disease. The secondary aim was to evaluate the associations of ferritin and other iron-related parameters with ADHD presentations and symptom severity within the ADHD group. To strengthen the interpretation of ferritin levels, hemoglobin and CRP were evaluated in both groups, and ferritin and CRP values obtained on the same day were used for analysis.

## Materials and methods

### Study design and setting

This retrospective cross-sectional study was conducted at Adana City Training and Research Hospital, a tertiary referral center in Adana, Türkiye. The study included children and adolescents who attended the Child and Adolescent Psychiatry and Pediatrics outpatient clinics between January 2025 and October 2025.

The study was designed to compare serum ferritin levels between non-anemic children and adolescents diagnosed with attention-deficit/hyperactivity disorder (ADHD) and a control group without known psychiatric or chronic systemic disease. In addition, the associations between ferritin levels, ADHD clinical presentations, and symptom severity were evaluated within the ADHD group.

Although the study included an ADHD group and a comparison group, it was not designed as a classical case-control study. Participants were not selected according to ferritin exposure or iron deficiency status, and no temporal exposure-outcome sequence could be established. Therefore, the study was defined as a retrospective cross-sectional analytical study with a comparison group to avoid implying causality.

### Participants

A total of 207 children and adolescents aged 6–18 years were included in the study. The ADHD group consisted of 105 patients who were diagnosed with ADHD according to the Diagnostic and Statistical Manual of Mental Disorders, Fifth Edition, Text Revision (DSM-5-TR) criteria. The control group consisted of 102 age-comparable children and adolescents without known psychiatric or chronic systemic disease who attended the Pediatrics outpatient clinics during the same period. Controls were recruited from the same hospital, calendar period, catchment area, and laboratory system as the ADHD group. Their outpatient visits were primarily for routine child health supervision, vaccination follow-up, school/sports health reports, or minor self-limited non-inflammatory complaints.

The ADHD diagnosis and clinical presentation were determined by child and adolescent psychiatrists based on clinical evaluation and DSM-5-TR diagnostic criteria. ADHD presentations were classified as predominantly inattentive presentation, predominantly hyperactive/impulsive presentation, or combined presentation.

### Inclusion criteria

The inclusion criteria for the ADHD group were as follows:

Children and adolescents aged 6–18 years, diagnosis of ADHD according to DSM-5-TR criteria, absence of anemia according to age- and sex-adjusted hemoglobin reference ranges, available hemoglobin, serum ferritin, and C-reactive protein values within the previous six months, and available Conners Parent and Teacher Rating Scale scores.

The inclusion criteria for the control group were as follows:

Children and adolescents aged 6–18 years, no known psychiatric diagnosis, no known chronic systemic disease, absence of anemia according to age- and sex-adjusted hemoglobin reference ranges, and available hemoglobin, serum ferritin, and C-reactive protein values within the previous six months.

### Exclusion criteria

Participants were excluded from both groups if they had anemia, active systemic disease, acute infection, chronic inflammatory disease, intellectual disability, hemoglobinopathy, chronic renal disease, chronic hepatic disease, or any known disorder that could substantially affect iron metabolism.

Participants with laboratory findings suggestive of acute inflammation were also excluded. Acute inflammation was evaluated using CRP levels obtained on the same day as ferritin measurement. In addition, individuals with incomplete essential clinical or laboratory data were not included in the final analysis.

### Data collection

Data were obtained retrospectively from the hospital information management system and patient records. Demographic, clinical, and laboratory variables were recorded.

The following variables were collected for all participants: age, sex, hemoglobin level, serum ferritin level, and C-reactive protein level. For the ADHD group, additional variables included ADHD clinical presentation, pharmacotherapy status, vitamin or mineral supplementation, serum iron level, total iron-binding capacity, transferrin saturation, and Conners Parent and Teacher Rating Scale scores.

Hemoglobin values were available for both the ADHD and control groups and were reviewed to exclude anemia according to age- and sex-adjusted reference ranges. However, detailed iron-related parameters other than ferritin, including serum iron, total iron-binding capacity, and transferrin saturation, were available only for the ADHD group. Therefore, between-group laboratory comparisons were performed for hemoglobin, serum ferritin, and C-reactive protein, while other iron-related parameters were evaluated only within the ADHD group.

When more than one laboratory result was available within the study period, the values closest to the psychiatric evaluation date were used. Serum ferritin and CRP values obtained on the same day were included in the analysis to minimize the potential confounding effect of acute inflammation on ferritin levels.

The six-month retrospective window was used to maximize eligible records while prioritizing the closest available laboratory values. This interval was not used to infer temporality, and possible ferritin variability between laboratory sampling and psychological assessment was addressed as a limitation.

### Laboratory measurements

Serum ferritin, serum iron, total iron-binding capacity, transferrin saturation, hemoglobin, and C-reactive protein values were obtained from routine laboratory results recorded in the hospital information management system.

Ferritin was used as a peripheral marker of iron stores. Because ferritin is also an acute-phase reactant, CRP was evaluated to account for the potential confounding effect of acute inflammation. Only serum ferritin and CRP values measured on the same day were used for analysis. Transferrin saturation was calculated or recorded according to the laboratory data available in the hospital system.

Anemia was defined according to age- and sex-adjusted hemoglobin reference ranges. Participants with anemia were excluded from both groups to allow evaluation of serum ferritin levels independent of overt anemia.

All laboratory data were obtained from routine results recorded in the same institutional central laboratory system. Serum ferritin concentrations were measured using an electrochemiluminescence immunoassay method with the Elecsys Ferritin assay kit (Roche Diagnostics, Mannheim, Germany) on the Elecsys 2010 analyzer platform, according to the manufacturer’s instructions. Hemoglobin, serum iron, total iron-binding capacity, transferrin saturation, and CRP were measured or recorded as part of the routine hospital laboratory workflow. Serum iron and other iron-related parameters were measured in morning fasting blood samples.

### Assessment of ADHD symptom severity

ADHD symptom severity was assessed using the Turkish validated versions of the Conners Parent and Teacher Rating Scales (14). Parent- and teacher-rated Conners scale scores were obtained from patient records. Hyperactivity scores and total symptom scores were recorded when available, with higher scores indicating greater symptom severity. The associations between serum ferritin levels and Conners scale scores were analyzed within the ADHD group.

### ADHD clinical presentations and treatment characteristics

ADHD clinical presentations were classified according to DSM-5-TR criteria as predominantly inattentive presentation, predominantly hyperactive/impulsive presentation, or combined presentation.

Current pharmacotherapy status was recorded from patient files. Treatment categories were defined as stimulant monotherapy (methylphenidate), non-stimulant monotherapy (atomoxetine), stimulant plus non-stimulant therapy (methylphenidate plus atomoxetine), stimulant plus antipsychotic adjunctive therapy (methylphenidate plus aripiprazole or risperidone), and no current ADHD medication. Vitamin or mineral supplementation within the previous six months was also recorded to evaluate its potential confounding effect on iron parameters. Proton pump inhibitor use was also reviewed because of its potential effect on iron absorption.

### Statistical analysis

Statistical analyses were performed using IBM SPSS Statistics version 26. The normality of continuous variables was assessed using the Shapiro–Wilk test. Since most continuous variables were not normally distributed, non-parametric tests were used.

Continuous variables were presented as median and interquartile range. Categorical variables were presented as frequencies and percentages. The Mann–Whitney U test was used to compare continuous variables between the ADHD and control groups. Categorical variables were compared using the chi-square test or Fisher’s exact test, as appropriate.

Within the ADHD group, laboratory parameters were compared across ADHD clinical presentations using the Kruskal–Wallis test. When a statistically significant difference was detected, pairwise comparisons were performed with correction for multiple comparisons.

Spearman correlation analysis was used to evaluate the relationships between serum ferritin levels and Conners Parent and Teacher Rating Scale scores.

Multivariable logistic regression analysis was performed to assess the association between serum ferritin levels and ADHD status after adjustment for age, sex, hemoglobin, and C-reactive protein. Odds ratios and 95% confidence intervals were calculated. ADHD status was coded as 1 for patients with ADHD and 0 for controls. Female sex was used as the reference category.

Ferritin was evaluated as a continuous variable in the main regression model. The regression model was used to assess association rather than causality because of the retrospective cross-sectional design. A p value of <0.05 was considered statistically significant.

No *a priori* sample size calculation was performed because this was a retrospective study including all eligible records from the predefined study period. BMI and standardized nutritional data were not consistently available in the retrospective records and therefore could not be included in regression models. Medication status was not included in the main ADHD-status logistic regression because ADHD medication use was applicable only to the ADHD group. To address symptom severity, exploratory linear regression analyses were performed within the ADHD group with parent- and teacher-rated hyperactivity scores as dependent variables and ferritin as the main predictor, adjusting for current ADHD medication use (yes/no) and vitamin/mineral supplementation.

### Ethical considerations

The study was conducted in accordance with the principles of the Declaration of Helsinki. Ethical approval was obtained from the Adana City Training and Research Hospital Scientific Research Ethics Committee, Adana, Türkiye. The ethics committee approved the study at meeting number 21, dated January 15, 2026, with decision number 1033. Because of the retrospective design of the study and the use of anonymized hospital records, the requirement for written informed consent was waived by the ethics committee. The confidentiality of all participant data was maintained throughout the study.

## Results

### Demographic and laboratory characteristics

A total of 207 children and adolescents were included in the study, comprising 105 patients with ADHD and 102 controls. There were no missing data for age, sex, hemoglobin, serum ferritin, or CRP. Serum ferritin and CRP values included in the analysis were obtained on the same day. Serum iron, total iron-binding capacity, and transferrin saturation values were available only for the ADHD group; therefore, between-group comparisons for these parameters could not be performed.

Most continuous variables were not normally distributed according to the Shapiro–Wilk test; therefore, non-parametric tests were used. The ADHD and control groups were comparable in terms of age, hemoglobin, and CRP levels. The median age was 10.0 years in both groups [ADHD: 10.0 years, interquartile range (IQR): 8.0–12.0; control: 10.0 years, IQR: 7.0–15.0; p=0.859]. Hemoglobin levels did not differ significantly between the ADHD and control groups [12.90 g/dL, IQR: 12.30–13.30 vs. 12.85 g/dL, IQR: 12.20–13.53; p=0.536]. Similarly, CRP levels were comparable between groups [2.00, IQR: 1.40–2.45 vs. 2.00, IQR: 1.20–2.40; p=0.931].

**Figure 1:**
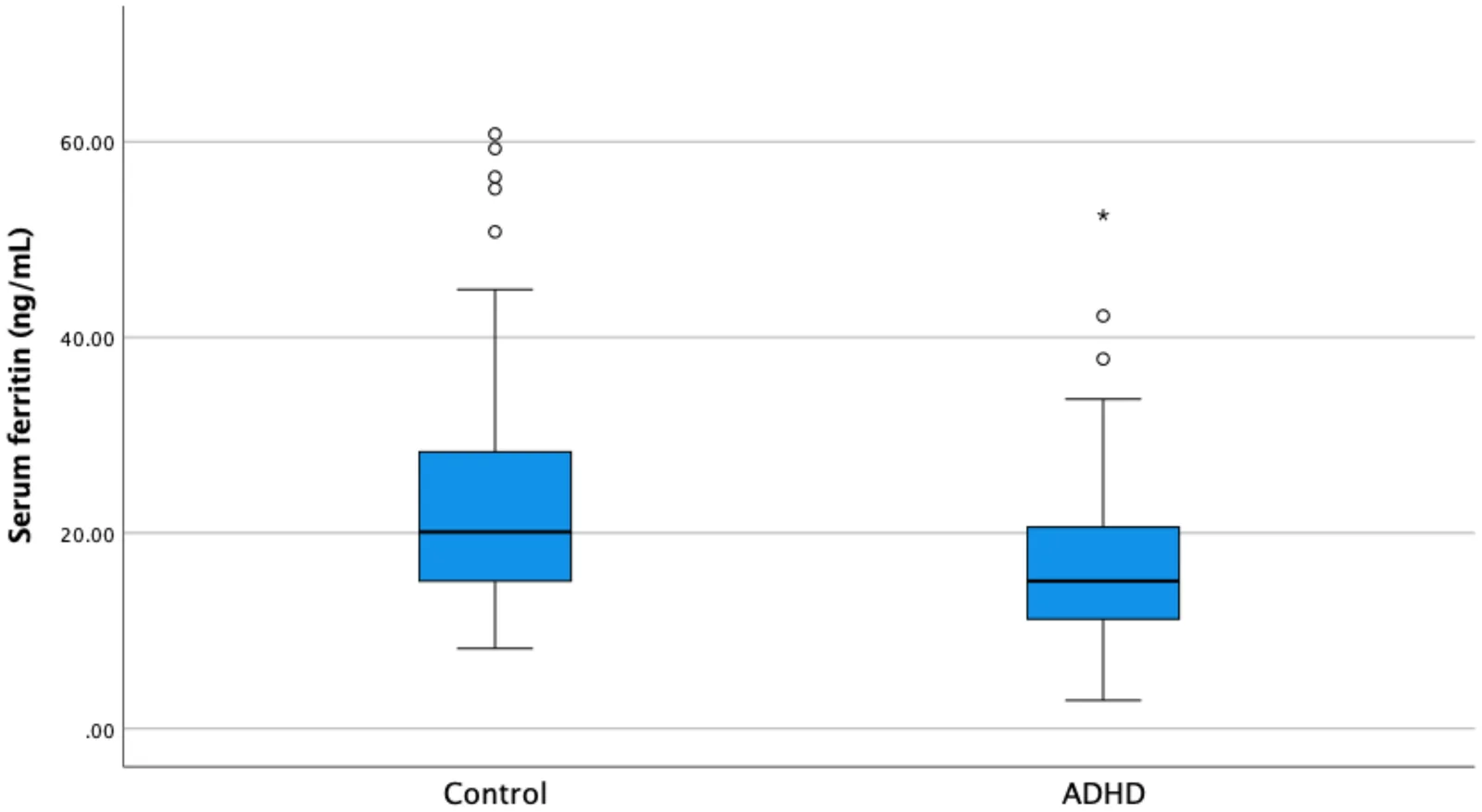
Comparison of serum ferritin levels between the ADHD and control groups. Children and adolescents with ADHD had significantly lower serum ferritin levels than controls (p<0.001). Boxplots represent the median, interquartile range, whiskers, and outliers. ADHD, attention-deficit/hyperactivity disorder.

The proportion of males was significantly higher in the ADHD group than in the control group [77/105 (73.3%) vs. 49/102 (48.0%); χ²=13.898, p<0.001]. Serum ferritin levels were significantly lower in the ADHD group compared with the control group [15.10 ng/mL, IQR: 11.10–20.60 vs. 20.10 ng/mL, IQR: 15.03–28.33; U = 3470.500, Z=-4.374, p<0.001] (**Table 1**) (**Figure 1**).

**Table 1:** Demographic and laboratory characteristics of the ADHD and control groups.

Variable	ADHD group n=105	Control group n=102	p value
Age, years, median [IQR]	10.0 [8.0-12.0]	10.0 [7.0-15.0]	0.859
Male sex, n (%)	77 (73.3)	49 (48.0)	<0.001
Hemoglobin, g/dL, median [IQR]	12.90 [12.30-13.30]	12.85 [12.20-13.53]	0.536
Ferritin, ng/mL, median [IQR]	15.10 [11.10-20.60]	20.10 [15.03-28.33]	<0.001
CRP, mg/L, median [IQR]	2.00 [1.40-2.45]	2.00 [1.20-2.40]	0.931

Continuous variables are presented as median [interquartile range]. Categorical variables are presented as n (%). Mann–Whitney U test was used for continuous variables, and Pearson chi-square test was used for categorical variables. Ferritin and CRP values were obtained on the same day.

### Clinical characteristics of the ADHD group

Among the 105 patients with ADHD, the most common clinical presentation was combined presentation, observed in 62 patients (59.0%). Predominantly inattentive presentation was present in 40 patients (38.1%), whereas predominantly hyperactive/impulsive presentation was observed in 3 patients (2.9%). Because the predominantly hyperactive/impulsive subgroup included only three patients, subgroup findings involving this presentation should be interpreted cautiously.

Regarding pharmacotherapy, 52 patients (49.5%) were receiving stimulant monotherapy with methylphenidate, 8 patients (7.6%) were receiving non-stimulant monotherapy with atomoxetine, 10 patients (9.5%) were receiving combined stimulant plus non-stimulant therapy, 28 patients (26.7%) were receiving stimulant plus antipsychotic adjunctive therapy with aripiprazole or risperidone, and 7 patients (6.7%) were not receiving current ADHD medication (**Table 2**).

**Table 2:** Clinical characteristics of the ADHD group.

Variable	ADHD group n=105
Predominantly inattentive presentation	40 (38.1)
Predominantly hyperactive/impulsive presentation	3 (2.9)
Combined presentation	62 (59.0)
Stimulant monotherapy (methylphenidate)	52 (49.5)
Non-stimulant monotherapy (atomoxetine)	8 (7.6)
Stimulant plus non-stimulant therapy (methylphenidate plus atomoxetine)	10 (9.5)
Stimulant plus antipsychotic adjunctive therapy (methylphenidate plus aripiprazole/risperidone)	28 (26.7)
No current ADHD medication	7 (6.7)
Vitamin/mineral supplementation within previous six months	31 (29.5)
No recorded vitamin/mineral supplementation	74 (70.5)
Proton pump inhibitor use	0 (0.0)

Values are presented as n (%). Percentages for pharmacotherapy categories were calculated within the ADHD group only (n=105). Stimulant treatment refers to methylphenidate, non-stimulant treatment refers to atomoxetine, and antipsychotic adjunctive therapy included aripiprazole or risperidone.

### Ferritin levels according to ADHD clinical presentations

Serum ferritin levels differed significantly across ADHD clinical presentations (p=0.001). The lowest median ferritin level was observed in the combined presentation group [13.50 ng/mL, IQR: 9.90–18.00]. The predominantly inattentive presentation group had a higher median ferritin level [19.20 ng/mL, IQR: 14.40–25.10]. The predominantly hyperactive/impulsive presentation group had the highest median ferritin level [33.70 ng/mL, IQR: 22.70–38.00]; however, this subgroup included only three patients.

Pairwise comparisons showed that the significant difference was mainly driven by lower ferritin levels in the combined presentation group compared with the predominantly inattentive presentation group (p=0.001). No statistically significant difference was observed between the predominantly inattentive and predominantly hyperactive/impulsive groups (p=0.252), or between the predominantly hyperactive/impulsive and combined presentation groups (p=0.101). Because of the very small number of patients in the predominantly hyperactive/impulsive group, ferritin differences across ADHD presentations should be interpreted as exploratory (**Table 3**).

**Table 3:** Serum ferritin levels according to ADHD clinical presentations.

ADHD presentation	n	Ferritin, ng/mL, median [IQR]
Predominantly inattentive	40	19.20 [14.40-25.10]
Predominantly hyperactive/impulsive	3	33.70 [22.70-38.00]
Combined	62	13.50 [9.90-18.00]

Overall p=0.001 by Kruskal–Wallis test.

Values are presented as median [interquartile range]. Pairwise comparisons showed a significant difference between the predominantly inattentive and combined presentation groups. The predominantly hyperactive/impulsive subgroup included only three patients; therefore, subgroup comparisons should be interpreted cautiously.

### Correlation between ferritin levels and symptom severity

Spearman correlation analysis was performed to evaluate the relationship between serum ferritin levels and ADHD symptom severity within the ADHD group. Serum ferritin levels showed weak negative correlations with parent-rated hyperactivity scores (r=-0.218, p=0.026) and teacher-rated hyperactivity scores (r=-0.217, p=0.026). No significant correlations were observed between serum ferritin levels and total Conners scores or other symptom domains, including inattentive symptoms.

These findings suggest that lower ferritin levels were weakly associated with higher hyperactivity scores; however, ferritin was not broadly associated with overall ADHD symptom burden. In exploratory within-ADHD linear regression analyses adjusted for current ADHD medication use and vitamin/mineral supplementation, ferritin remained associated with teacher-rated hyperactivity (B=-0.152, p=0.027) and showed a borderline association with parent-rated hyperactivity (B=-0.070, p=0.061). These findings indicate that the unadjusted correlations should be interpreted cautiously, particularly for parent-rated symptoms.

### Multivariable logistic regression analysis

A multivariable logistic regression analysis was performed to evaluate the association between serum ferritin levels and ADHD status after adjustment for age, sex, hemoglobin, and CRP. ADHD status was coded as 1 for patients with ADHD and 0 for controls. The overall model was statistically significant (χ²=37.508, df=5, p<0.001), with a Cox and Snell R² of 0.166, a Nagelkerke R² of 0.221, and an overall classification accuracy of 69.6%.

Higher serum ferritin levels were significantly associated with lower odds of ADHD after adjustment for age, sex, hemoglobin, and CRP (OR = 0.927, 95% CI: 0.895–0.961; p<0.001). Male sex was also significantly associated with higher odds of ADHD (OR = 3.335, 95% CI: 1.783–6.235; p<0.001). Age, hemoglobin, and CRP were not significantly associated with ADHD status in the adjusted model (**Table 4**).

**Table 4:** Multivariable logistic regression analysis for ADHD status.

Variable	B	SE	Wald	OR	95% CI	p value
Ferritin, ng/mL	-0.076	0.018	17.111	0.927	0.895-0.961	<0.001
Age, years	-0.028	0.048	0.327	0.973	0.885-1.069	0.567
Male sex	1.204	0.319	14.224	3.335	1.783-6.235	<0.001
Hemoglobin, g/dL	-0.235	0.150	2.468	0.791	0.590-1.060	0.116
CRP, mg/L	-0.002	0.141	0.000	0.998	0.756-1.316	0.988

ADHD status was coded as 1 for patients with ADHD and 0 for controls. Female sex was used as the reference category. The model was used to assess association rather than causality.

## Discussion

In this retrospective cross-sectional study, non-anemic children and adolescents with ADHD had significantly lower serum ferritin levels than controls without known psychiatric or chronic systemic disease, whereas hemoglobin and CRP levels were comparable between the two groups. In addition, higher ferritin levels were associated with lower odds of ADHD after adjustment for age, sex, hemoglobin, and CRP. Within the ADHD group, ferritin levels differed across ADHD presentations and showed weak negative correlations with parent- and teacher-rated hyperactivity scores. These findings suggest a possible association between lower peripheral iron stores and ADHD-related clinical features, but they do not establish causality.

The study design should be interpreted as cross-sectional and analytical rather than classical case-control. Although ADHD and control groups were compared, ferritin status was not used as an exposure-defining variable, and no temporal relationship between iron status and ADHD onset could be established. Therefore, the observed association between lower ferritin levels and ADHD status should be interpreted as an association within a retrospective cross-sectional framework, not as evidence that low ferritin precedes or causes ADHD.

The finding of lower ferritin levels in the ADHD group is consistent with previous evidence suggesting that peripheral iron stores may be reduced in children with ADHD. A systematic review and meta-analysis by Tseng et al. reported significantly lower serum ferritin levels in children with ADHD compared with controls, although findings for serum iron and other peripheral iron parameters were less consistent (8). Similarly, Wang et al. suggested that altered iron status may be associated with ADHD, particularly when ferritin is used as a marker of iron storage (9).

From a biological perspective, this association is plausible. Iron is involved in dopamine synthesis, dopamine metabolism, myelination, mitochondrial function, and neuronal development. Since dopaminergic dysfunction has been implicated in core ADHD symptoms, reduced iron stores may contribute to symptom expression in a subset of children and adolescents with ADHD (5, 6). However, ferritin is a peripheral biomarker and does not directly reflect brain iron concentration, which limits the interpretation of serum ferritin as a direct indicator of central nervous system iron status (15). Therefore, serum ferritin should be considered an indirect marker of peripheral iron stores rather than a direct measure of brain iron availability.

The relationship between ferritin and ADHD is unlikely to be simple or linear. Percinel et al. did not find a significant difference in ferritin levels between children and adolescents with ADHD and controls, nor did they observe a significant association between ferritin levels and symptom severity (11). Tang and Wen also reported that serum ferritin levels may vary across children with ADHD and tic disorder, suggesting that ferritin-related alterations may not be specific to ADHD alone (16). Differences in study design, sample characteristics, comorbidities, inflammatory status, dietary factors, and medication exposure may partly explain these inconsistent findings.

In the present study, ferritin levels were lowest in the combined presentation group. This finding may suggest that reduced iron stores are more closely related to broader or more severe ADHD symptom profiles. Nevertheless, this result should be interpreted cautiously because the predominantly hyperactive/impulsive presentation group included only three patients. Therefore, ferritin should not be considered a reliable marker for distinguishing ADHD presentations based on the present data alone. Previous studies evaluating iron-related parameters across ADHD presentations have also reported heterogeneous findings, supporting the possibility that the relationship between ferritin and ADHD phenotype may vary across clinical subgroups (11). Accordingly, the subgroup analysis in this study should be regarded as exploratory.

The weak negative correlations between ferritin levels and both parent- and teacher-rated hyperactivity scores may be clinically informative but should be interpreted conservatively. These findings suggest that ferritin may be more closely associated with hyperactivity than with inattentive symptoms or total symptom burden. Okuyucu et al. similarly reported that lower ferritin levels were associated with greater ADHD symptom severity and poorer daily life skills in children with ADHD (10). However, the correlation coefficients in our study were low, indicating that ferritin explains only a small proportion of symptom variability. Thus, ferritin alone is unlikely to capture the multidimensional clinical severity of ADHD.

The persistence of the ferritin difference between the ADHD and control groups after adjustment for age, sex, hemoglobin, and CRP strengthens the observation that lower ferritin levels may be associated with ADHD independent of basic demographic, hematologic, and inflammatory variables. This is important because ferritin is an acute-phase reactant and may be affected by inflammation, infection, chronic disease, and nutritional status (7). In the present study, similar CRP levels between groups reduce, but do not completely eliminate, the possibility that inflammation influenced ferritin levels. The use of ferritin and CRP values measured on the same day further strengthens the interpretation of ferritin levels in relation to inflammatory status.

Another important aspect of this study is the inclusion of non-anemic participants. This allowed the evaluation of iron stores independently of overt anemia. Previous research has suggested that neurobehavioral effects of low iron stores may occur even in the absence of anemia, particularly in neurodevelopmental conditions where dopamine-related pathways are implicated (6, 7). Therefore, assessing ferritin levels in non-anemic children with ADHD may provide clinically meaningful information beyond routine hemoglobin evaluation. However, the clinical relevance of low-normal ferritin levels in ADHD requires prospective confirmation.

The clinical implications of these findings should be considered carefully. The present results do not support the use of ferritin as a diagnostic biomarker for ADHD. Rather, ferritin may be considered a supportive biological parameter in selected patients, especially those with more prominent hyperactivity symptoms or clinical features suggestive of low iron stores. Recent evidence on iron supplementation in neurodevelopmental disorders suggests potential benefits in selected populations, but the available data remain heterogeneous and insufficient to support routine supplementation without biochemical evidence of deficiency (17). Similarly, recent reviews on serum ferritin and ADHD have emphasized that ferritin may be biologically relevant but should not be interpreted as a standalone biomarker because the current evidence remains heterogeneous (18).

Therefore, iron supplementation should not be inferred from the present findings alone. In this manuscript, biochemical evidence of iron deficiency refers primarily to low serum ferritin interpreted together with age, hemoglobin level, inflammatory status, and local laboratory reference ranges. According to WHO guidance, serum ferritin <15 micrograms/L indicates iron deficiency in apparently healthy children aged 5 to <10 years and adolescents aged 10 to <20 years, whereas higher thresholds may be required in the presence of infection or inflammation. Accordingly, supplementation should be considered only after individualized clinical evaluation and biochemical confirmation of deficiency, rather than on the basis of ADHD diagnosis or symptom severity alone (19).

Sleep parameters also deserve attention in future studies evaluating ferritin and ADHD. Non-anemic iron deficiency and reduced central nervous system iron availability have been implicated in sleep-related movement disorders such as restless legs syndrome, periodic limb movements during sleep, and restless sleep disorder. These conditions are clinically relevant in ADHD because sleep disruption may worsen daytime attention, hyperactivity, emotional regulation, and overall functioning. Future studies should include standardized sleep histories, screening for restless legs symptoms, and, when clinically indicated, objective sleep assessments (20, 21).

This study has several limitations. First, its retrospective, cross-sectional, and single-center design limits causal interpretation and generalizability. Second, serum ferritin is a peripheral marker of iron stores and does not directly reflect brain iron availability. Third, although hemoglobin and CRP were evaluated in both groups and ferritin and CRP values measured on the same day were used, other factors that may influence ferritin levels, including dietary iron intake, socioeconomic status, recent infections, sleep patterns, medication duration, comorbid psychiatric conditions, family history, BMI, and vitamin or mineral supplementation, could not be fully controlled. BMI and standardized nutritional data were not consistently available in the retrospective records, and vitamin/mineral supplementation was recorded for ADHD participants but not systematically for controls; therefore, these variables could not be included in the main regression model. Medication-adjusted symptom analyses were exploratory because medication status was applicable only within the ADHD group. Laboratory values obtained within a six-month retrospective window were eligible, and ferritin may vary during this interval, even though the closest available values and same-day ferritin/CRP measurements were selected. No *a priori* sample size calculation was performed; instead, all eligible records from the predefined study period were included. Although sex was included in the multivariable regression model, the unequal sex distribution between the ADHD and control groups may still represent a potential source of residual confounding. Serum iron, total iron-binding capacity, and transferrin saturation values were available only within the ADHD group; therefore, between-group comparisons for these parameters could not be performed. Finally, the predominantly hyperactive/impulsive subgroup included only three patients, limiting the reliability of subgroup comparisons across ADHD presentations. Therefore, findings related to ADHD presentations should be interpreted as exploratory.

Despite these limitations, the study has several strengths. The exclusion of anemic participants allowed the assessment of ferritin as an indicator of iron stores independent of overt anemia. The inclusion of hemoglobin and CRP in both group comparisons and regression analyses strengthened the interpretation of ferritin differences independent of overt anemia and inflammation. Another strength is that laboratory data were obtained from the same institutional laboratory system, and ferritin was interpreted together with same-day CRP, reducing heterogeneity related to assay source and acute inflammation. ADHD symptom severity was evaluated using both parent and teacher reports, providing information from different functional settings. Future prospective studies should evaluate ferritin together with dietary intake, inflammatory markers, treatment exposure, sleep parameters, and neurodevelopmental comorbidities to better clarify the role of iron metabolism in ADHD (4, 17).

## Conclusion

This study demonstrated that non-anemic children and adolescents with ADHD had significantly lower serum ferritin levels than controls without known psychiatric or chronic systemic disease, despite comparable hemoglobin and CRP levels. Lower ferritin levels were associated with ADHD status after adjustment for age, sex, hemoglobin, and CRP. Ferritin levels were also weakly associated with hyperactivity scores and differed across ADHD presentations, particularly between combined and predominantly inattentive presentations. However, serum ferritin should be interpreted as a supportive biological parameter rather than a diagnostic or severity biomarker. These findings should be considered exploratory because of the retrospective cross-sectional design, unequal sex distribution, small subgroup sizes, absence of an *a priori* sample size calculation, possible temporal variability between laboratory and psychological assessments, and incomplete data on BMI, diet, medication duration, supplementation in controls, and sleep parameters. Prospective, multicenter studies controlling for dietary intake, inflammation, BMI, medication exposure, supplement use, sleep characteristics, and neurodevelopmental comorbidities are needed to clarify the role of iron metabolism in ADHD.

## Data Availability

The original contributions presented in the study are included in the article/supplementary material. Further inquiries can be directed to the corresponding author.
